# Heterogeneity of metabolic adaptive capacity affects the prognosis among pancreatic ductal adenocarcinomas

**DOI:** 10.1007/s00535-022-01898-0

**Published:** 2022-07-03

**Authors:** Taishu Kanda, Taiichi Wakiya, Keinosuke Ishido, Norihisa Kimura, Hiroaki Fujita, Tadashi Yoshizawa, Shintaro Goto, Yota Tatara, Hiroshi Kijima, Kenichi Hakamada

**Affiliations:** 1 Department of Gastroenterological Surgery, Hirosaki University Graduate School of Medicine, 5 Zaifu-cho, Hirosaki, Aomori 036-8562 Japan; 2 Department of Pathology and Bioscience, Hirosaki University Graduate School of Medicine, Hirosaki, Aomori 036-8562 Japan; 3 Department of Stress Response Science, Center for Advanced Medical Research, Hirosaki University Graduate School of Medicine, Hirosaki, Aomori 036-8562 Japan

**Keywords:** Biological evolution, CA19-9, Heterogeneity, Metabolic reprogramming, Pancreatic cancer

## Abstract

**Background:**

Evolutionary cancer has a supply mechanism to satisfy higher energy demands even in poor-nutrient conditions. Metabolic reprogramming is essential to supply sufficient energy. The relationship between metabolic reprogramming and the clinical course of pancreatic ductal adenocarcinoma (PDAC) remains unclear. We aimed to clarify the differences in metabolic status among PDAC patients.

**Methods:**

We collected clinical data from 128 cases of resectable PDAC patients undergoing surgery. Sixty-three resected tissues, 15 tissues from the low carbohydrate antigen 19-9 (CA19-9), 38–100 U/mL, and high CA19-9, > 500 U/mL groups, and 33 non-tumor control parts, were subjected to tandem mass spectrometry workflow to systematically explore metabolic status. Clinical and proteomic data were compared on the most used PDAC biomarker, preoperative CA19-9 value.

**Results:**

Higher CA19-9 levels were clearly associated with higher early recurrence (*p* < 0.001), decreased RFS (*p* < 0.001), and decreased DSS (*p* = 0.025). From proteomic analysis, we discovered that cancer evolution-related as well as various metabolism-related pathways were more notable in the high group. Using resected tissue immunohistochemical staining, we learned that high CA19-9 PDAC demonstrated aerobic glycolysis enhancement, yet no decrease in protein synthesis. We found a heterogeneity of various metabolic processes, including carbohydrates, proteins, amino acids, lipids, and nucleic acids, between the low and the high groups, suggesting differences in metabolic adaptive capacity.

**Conclusions:**

Our study found metabolic adaptation differences among PDAC cases, pertaining to both cancer evolution and the prognosis. CA19-9 can help estimate the metabolic adaptive capacity of energy supply for PDAC evolution.

**Supplementary Information:**

The online version contains supplementary material available at 10.1007/s00535-022-01898-0.

## Introduction

Pancreatic ductal adenocarcinoma (PDAC) is the most lethal cancer, and there is a worldwide need to improve the prognosis [1, 2]. PDAC has particularly high levels of carbohydrate antigen 19-9 (CA19-9) resulting in an extremely poor prognosis [3]. Radical surgery is the only treatment offering a hope of cure for patients with PDAC. Unfortunately, distant metastasis and postoperative recurrence are common in PDAC with high CA19-9 levels [3]. Thus, novel therapeutic strategies are necessary to target this; however, the mechanisms of why metastasis and recurrence are more common in cases with high levels, compared to low CA19-9 PDAC, have not been fully elucidated.

CA19-9 has long been known as a biomarker and widely accepted as a predictor for PDAC [4]. Furthermore, several lines of evidence have shown that CA19-9 is functionally implicated in its malignant evolution by affecting metastasis through binding to selectin and facilitating angiogenesis [4–7]. In short, in PDAC with high CA19-9 levels, CA19-9 can function as a promoter, leading to further cancer evolution.

Generally, cancer evolution has higher energy demand because cancer cell growth and proliferation need sustained anabolic processes that require energy [8]. Paradoxically, evolutionary cancer must be equipped with a full energy supply even in hypoxia and hypo-nutrient conditions. In order for cancer cells to acquire sufficient energy supporting cell growth and proliferation, extensive metabolic rewriting must occur [9]. This metabolic adaptation is called metabolic reprogramming, which has been proposed as a hallmark of cancer cells [10]. These insights evoked the notion among us that the heterogeneity of metabolic reprogramming is involved in the acquisition of malignant evolution in PDAC. However, this association had not been adequately verified using clinical specimens. Therefore, in this study, we aimed to clarify whether there were differences in metabolic reprogramming between the low and high CA19-9 PDAC patients. Here, we present the heterogeneity of metabolic adaptation among cases of PDAC, which is reflected in the prognosis, accordingly.

## Methods

### Patients

This single-center, retrospective, observational study was approved by the Committee of Medical Ethics of Hirosaki University Graduate School of Medicine (reference no. 2020-002). Informed consent was obtained in the form of an opt-out option on our website (https://www.med.hirosaki-u.ac.jp/hospital/outline/resarch/resarch.html), with the approval of the Committee of Medical Ethics of Hirosaki University Graduate School of Medicine. This study was designed and carried out in accordance with the Declaration of Helsinki.

A total of 128 patients undergoing pancreatic surgery for resectable PDAC with, curative intent, at our facility between 2007 and 2018 were included in this study. All patients had a confirmed pathologic diagnosis. Resectability status was made based on the National Comprehensive Cancer Network guidelines. None of the included patients received neoadjuvant therapy before surgery.

### Surgical procedures and operative management

We selected the type of pancreatic resection based on the tumor location. Open pancreatoduodenectomy with lymph node dissections was usually performed for cases of pancreatic head cancer. In cases of pancreatic body and tail cancer, open or minimally invasive distal pancreatectomy was performed with lymph node dissections. If swelling of a paraaortic lymph node was detected, we generally performed paraaortic lymph node sampling during the pancreatoduodenectomy, whereas sampling was not routinely performed during distal pancreatectomy. We performed a fresh frozen section analysis to confirm whether or not the pancreatic cut-end margin was clear of residual cancer. If residual cancer was present at the pancreatic cut-end margin, we cut the pancreas further to reach negative margin status. If necessary, to achieve curative resection, we performed a total pancreatectomy with lymph node dissections.

### Histological grading of pancreatic cancers

All slides which were originally prepared from formalin-fixed and paraffin-embedded tissue were reviewed. Morphological analyses were performed using 4-µM slides stained with hematoxylin and eosin (H&E). Grading of histological findings of the resected pancreatic tissues was performed referring to an existing scoring system for pancreatic cancer [11, 12]. The slides were examined by board-certified pathologists unaware of the clinical data.

### Classification and comparison of patients

The 128 patients were divided into four groups, based on their preoperative CA19-9 values as follows; normal group (CA19-9: < 38 U/mL), low group (CA19-9: 38–100 U/mL), intermediate group (CA19-9: 101–500 U/mL), and high group (CA19-9: > 500 U/mL). These cutoff values were based on previous studies that verified the usefulness of CA19-9 as a biomarker [3, 13–16]. In comparing perioperative factors, the medical records for each case were reviewed and compared among the groups. All postoperative measurements of CA19-9 were taken within 2 months following surgery.

### Tissue section preparation

Supplemental Methods 1.

### Liquid chromatography with tandem mass spectrometry (LC–MS/MS)

Supplemental Methods 1.

### Proteomics data analysis

Supplemental Methods 1.

### Immunohistochemistry (IHC)

Supplemental Methods 1.

### Statistical analyses

Continuous variables were expressed as the medians (ranges) and analyzed using nonparametric methods for non-normally distributed data (Mann–Whitney *U* test). Categorical variables were reported as numbers (percentages) and analyzed using the Chi-squared test or Fisher’s exact test, as appropriate. Variables with a significant relationship with high CA19-9 levels in univariate analysis were used in a binary logistic regression analysis. Recurrence-free survival (RFS) and Disease-specific survival (DSS) were calculated using the Kaplan–Meier method, and differences in the survival rates among the groups were compared using the log-rank test. RFS was defined as the time from the operation to the date of disease recurrence. DSS was defined as the time from the operation to the time of death due to PDAC, or the last follow-up time. This study was planned with a maximum follow-up period of 5 years. A difference was considered to be significant for values of *p* < 0.05. The statistical analyses were performed using IBM SPSS Statistics for Windows, Version 26.0 (IBM Corp, Armonk, NY, USA).

## Results

### Comparison of the clinical characteristics and operation-related factors across the groups

First, we investigated the clinical characteristics and outcomes according to their preoperative CA19-9 values. Of the 128 patients, 46 (35.9%) were included in the normal group, 24 (18.8%) in the low group, 34 (26.6%) in the intermediate group, and 24 (18.8%) in the high group. A comparison of the clinical characteristics and operation-related factors between groups is shown in Table 1. There were also significant differences in tumor biomarker values other than CA19-9 between groups. There were no significant differences in operation-related factors across the groups. Consistently, postoperative CA19-9 values were significantly different between groups (*p* < 0.001).

**Table 1:** Comparison of the clinical characteristics and operation-related factors in the entire cohort

	All (*n* = 128)	Normal (*n* = 46)	Low (*n* = 24)	Intermediate (*n* = 34)	High (*n* = 24)	*p* value
Gender, male, *n*	65 (50.8)	21 (45.7)	11 (45.8)	18 (52.9)	15 (62.5)	0.552
Age, year	70 (50–85)	69 (50–85)	72 (54–79)	69 (52–80)	69 (52–82)	0.552
Body weight, kg	56.0 (34.0–85.0)	54.5 (36.0–85.0)	55.5 (38.5–76.6)	56.2 (34–85)	56.9 (34.7–82.5)	0.751
Body mass index, kg/m^2^	22.3 (14.1–36.3)	21.6 (16.4–36.3)	22.7 (16.2–28.3)	22.6 (14.1–30.2)	22.3 (15.8–33.3)	0.745
Obstructive jaundice, *n*	60 (46.9)	22 (47.8)	10 (41.7)	16 (47.1)	12 (50.0)	0.946
Diabetes mellitus, *n*	43 (33.6)	11 (23.9)	12 (50.0)	12 (35.3)	8 (33.3)	0.181
Laboratory values						
WBC, /µL	5165 (2230–11,020)	4965 (2340–11,020)	5490 (2230–7460)	5340 (2790–8320)	5200 (3980–8680)	0.189
Hemoglobin, g/dL	12.7 (7.2–16.3)	12.3 (7.2–15.8)	12.6 (9.9–14.9)	13.5 (8.8–16.3)	12.1 (10.4–14.7)	0.126
Hematocrit, %	37.2 (22.8–46.1)	36.7 (22.8–44.8)	37.3 (29.3–43.0)	40.1 (26.7–46.1)	36.3 (29.6–43.5)	0.104
Platelets, × 10^3^/µL	222 (64–513)	216 (96–513)	192 (96–393)	226 (100–404)	242 (64–358)	0.369
CRP, mg/dL	0.14 (0.02–9.59)	0.12 (0.02–6.50)	0.11 (0.02–7.30)	0.18 (0.02–9.59)	0.29 (0.02–5.40)	0.574
Albumin, g/dL	3.9 (2.0–5.7)	3.9 (2.4–4.9)	3.9 (2.4–5.7)	4.1 (2.0–5.0)	3.9 (2.5–4.6)	0.374
Total protein, g/dL	6.8 (4.9–8.9)	6.9 (5.0–7.9)	6.6 (5.4–8.9)	7.1 (4.9–7.9)	6.8 (5.3–8.1)	0.072
Creatinine, mg/dL	0.64 (0.40–1.43)	0.62 (0.40–1.00)	0.65 (0.46–1.10)	0.70 (0.46–1.43)	0.60 (0.43–1.30)	0.279
AST, U/L	29 (11–406)	27 (11–260)	27 (13–91)	35 (14–406)	52 (15–287)	0.089
ALT, U/L	35 (9–627)	26 (9–627)	28 (12–210)	42 (13–621)	73 (12–443)	0.082
GTP, U/L	65 (9–1720)	38 (9–713)	83 (16–529)	80 (12–1720)	104 (9–893)	0.251
Total bilirubin, mg/dL	0.8 (0.2–32.7)	0.7 (0.3–24.1)	0.8 (0.3–24.0)	0.8 (0.4–32.7)	1.7 (0.2–24.1)	0.155
Amylase, U/L	74 (17–737)	71 (31–737)	82 (35–462)	72 (21–167)	78 (17–231)	0.587
Total cholesterol	187 (84–653)	191 (112–653)	173 (129–258)	187 (109–361)	197 (84–563)	0.652
HbA1c, %	6.0 (4.4–11.9)	5.9 (4.4–11.3)	6.4 (4.8–9.8)	5.7 (4.4–11.8)	6.8 (4.4–11.9)	0.302
CA19-9, U/mL	71 (1–9675)	14 (1–36)	61 (38–96)	169 (105–481)	1401 (511–9675)	< 0.001
CEA, ng/mL	2.7 (0.5–37.0)	2.1 (0.5–10.1)	2.8 (0.7–37.0)	3.3 (0.9–23.9)	4.3 (1.0–17.0)	0.001
DUPAN, U/mL	96 (22–16,000)	36 (25–16,000)	48 (22–625)	169 (27–624)	523 (31–10,800)	< 0.001
SPAN, U/mL	51 (2–2284)	17 (2–2284)	38 (24–142)	68 (33–161)	496 (153–1667)	< 0.001
Operative outcomes						
Procedure, *n*						0.574
Pancreaticoduodenectomy	83 (64.8)	31 (67.4)	12 (50.0)	22 (64.7)	18 (75.0)	
Distal pancreatectomy	40 (31.3)	13 (28.3)	10 (41.7)	11 (32.4)	6 (25.0)	
Total pancreatectomy	5 (3.9)	2 (4.3)	2 (8.3)	1 (2.9)	0	
Operation time, min	310 (91–647)	309 (91–647)	294 (139–573)	323 (115–587)	310 (130–513)	0.843
Intraoperative blood loss, mL	765 (90–3915)	710 (90–2450)	775 (155–2270)	885 (150–2775)	1003 (200–3915)	0.202
Intraoperative ABT	24 (18.8)	5 (10.9)	5 (20.8)	7 (20.6)	7 (29.2)	0.292
Portal vein resection, *n*	19 (14.8)	7 (15.2)	2 (8.3)	5 (14.7)	5 (20.8)	0.684
Postoperative CA19-9, U/mL	18 (1–1065)	8 (1–19)	16 (6–33)	41 (13–193)	147.5 (9–1065)	< 0.001
CA19-9 elevation after surgery, *n*	6 (4.7)	5 (10.9)^a^	0	1 (2.9)^b^	0	0.089

*ABT* allogeneic red blood cell transfusion, *CA19-9* carbohydrate antigen 19-9, *CEA* carcinoembryonic antigen, *DUPAN* duke pancreatic monoclonal antigen, *SPAN* s-pancreas antigen

^a^All the postoperative values were within the recommended upper limit of normal

^b^The patient showed an early hepatic recurrence after surgery

### The high group shows a larger tumor size across the groups

A comparison of the pathological characteristics between groups is shown in Table 2. There were significant differences in maximum tumor size among the groups. The maximum tumor size in the high group was the largest. Moreover, the high group, by and large, showed a greater prevalence of local invasion factors. On the other hand, the prevalence of portal vein and artery invasion was similar from group to group. This result seemed logical, because the current study included only resectable PDAC cases.

**Table 2:** Comparison of the pathological characteristics in the entire cohort

	All (*n* = 128)	Normal (*n* = 46)	Low (*n* = 24)	Intermediate (*n* = 34)	High (*n* = 24)	*p* value
Tumor size, mm	30 (7–150)	28 (7–130)	30 (11–150)	34 (15–60)	39 (25–70)	0.019
UICC 8th edition						
T category, *n*						0.100
T1	16 (12.5)	8 (17.4)	2 (8.3)	6 (17.6)	0	
T2	79 (61.7)	29 (63.0)	15 (62.5)	22 (64.7)	13 (54.2)	
T3	33 (25.8)	9 (19.6)	7 (29.2)	6 (17.6)	11 (45.8)	
T4	0	0	0	0	0	
N category, *n*						0.245
N0	49 (38.3)	21 (45.7)	13 (54.2)	9 (26.5)	6 (25.0)	
N1	49 (38.3)	16 (34.8)	6 (25.0)	15 (44.1)	12 (50.0)	
N2	30 (23.4)	9 (19.6)	5 (20.8)	10 (29.4)	6 (25.0)	
M category, *n*						0.824
M0	117 (91.4)	41 (89.1)	22 (91.7)	31 (91.2)	23 (95.8)	
M1^a^	11 (8.6)	5 (10.9)	2 (8.3)	3 (8.8)	1 (4.2)	
UICC stage, *n*						0.371
IA	12 (9.4)	8 (17.4)	2 (8.3)	2 (5.9)	0	
IB	24 (18.8)	8 (17.4)	7 (29.2)	6 (17.6)	3 (12.5)	
IIA	12 (9.4)	4 (8.7)	4 (16.7)	1 (2.9)	3 (12.5)	
IIB	45 (35.2)	14 (30.4)	5 (20.8)	14 (41.2)	12 (50.0)	
III	24 (18.8)	7 (15.2)	4 (16.7)	8 (23.5)	5 (20.8)	
IV	11 (8.6)	5 (10.9)	2 (8.3)	3 (8.8)	1 (4.2)	
R0 resection, *n*	114 (89.1)	37 (80.4)	24 (100.0)	32 (94.1)	21 (87.5)	0.060
Local invasion factor						
Bile duct invasion	60 (46.9)	22 (47.8)	10 (41.7)	16 (47.1)	12 (50.0)	0.946
Duodenal invasion	56 (43.8)	16 (34.8)	8 (33.3)	15 (44.1)	17 (70.8)	0.021
Serosal side of the anterior pancreatic tissue invasion	28 (21.9)	10 (21.7)	4 (16.7)	9 (26.5)	5 (20.8)	0.845
Retropancreatic tissue invasion	103 (80.5)	35 (76.1)	18 (75.0)	29 (85.3)	21 (87.5)	0.517
Portal venous system invasion	28 (21.9)	9 (19.6)	5 (20.8)	10 (29.4)	4 (16.7)	0.644
Arterial system invasion	20 (15.6)	7 (15.2)	4 (16.7)	6 (17.6)	3 (12.5)	0.958
Extrapancreatic nerve plexus invasion	28 (21.9)	9 (19.6)	6 (25.0)	8 (23.5)	5 (20.8)	0.950
Invasion of other organs	6 (4.7)	2 (4.3)	1 (4.2)	1 (3.0)	2 (8.3)	0.817
Assessment of TME						
Lymphatic invasion						0.087
No evidence of invasion	5 (3.9)	4 (8.7)	1 (4.2)	0	0	
Slight invasion	24 (18.8)	12 (26.1)	5 (20.8)	5 (14.7)	2 (8.3)	
Moderate invasion	59 (46.1)	13 (28.3)	12 (50.0)	21 (61.8)	13 (54.2)	
Marked invasion	40 (31.3)	17 (37.0)	6 (25.0)	8 (23.5)	9 (37.5)	
Venous invasion						0.193
No evidence of invasion	7 (5.5)	4 (8.7)	2 (8.3)	1 (2.9)	0	
Slight invasion	33 (25.8)	16 (34.8)	5 (20.8)	8 (23.5)	4 (16.7)	
Moderate invasion	58 (45.3)	19 (41.3)	14 (58.3)	14 (41.2)	11 (45.8)	
Marked invasion	30 (23.4)	7 (15.2)	3 (12.5)	11 (32.4)	9 (37.5)	
Nerve invasion						0.171
No evidence of invasion	5 (3.9)	4 (8.7)	1 (4.2)	0	0	
Slight invasion	15 (11.7)	6 (13.0)	5 (20.8)	3 (8.8)	1 (4.2)	
Moderate invasion	34 (26.6)	13 (28.3)	5 (20.8)	12 (35.3)	4 (16.7)	
Marked invasion	74 (57.8)	23 (50.0)	13 (54.2)	19 (55.9)	19 (79.2)	
Cancer–stroma relationship						0.511
Medullary type	4 (3.1)	1 (2.2)	2 (8.3)	1 (3.0)	0	
Intermediate type	74 (58.3)	30 (65.2)	12 (50.0)	20 (60.6)	12 (50.0)	
Scirrhous type	49 (38.6)	15 (32.6)	10 (41.7)	12 (36.4)	12 (50.0)	

*TME* tumor microenvironment, *UICC* Union for International Cancer Control

^a^All the patients were diagnosed with M1 due to positive lymph nodes other than the regional lymph nodes

### The high group shows a poor prognosis after radical surgery for resectable PDAC

**Fig. 1:**
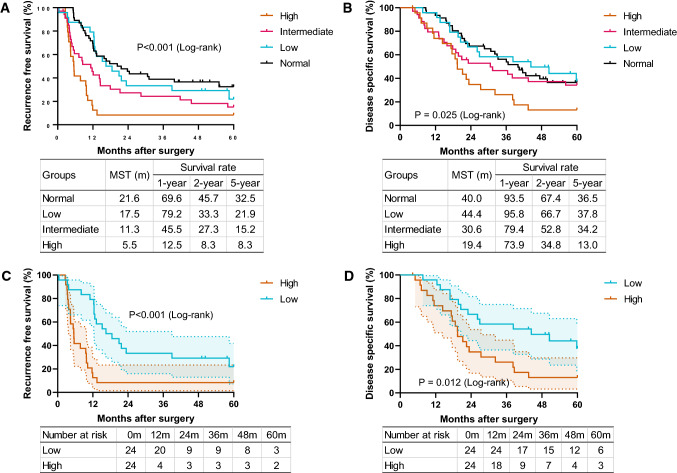
High CA19-9 levels are closely associated with poor prognoses in patients with PDAC. **A** Recurrence-free survival rates in resectable PDAC patients with normal (*n* = 46), low (*n* = 24), intermediate (*n* = 34), and high (*n* = 24) CA19-9 levels assessed by log-rank test (*p* < 0.001). **B** Disease-specific survival rates in resectable PDAC patients among the four groups (*p* = 0.025). **C** Recurrence-free survival rates in resectable PDAC patients with low (*n* = 24) or high (*n* = 24) CA19-9 levels assessed by log-rank test (*p* < 0.001). **D** Disease-specific survival rates in resectable PDAC patients between two groups (*p* = 0.012). The graph in **C** and **D** shows the staircase error envelopes that enclose the 95% confidence interval for the probability of survival

There were no significant differences in the incidences of short-term postoperative outcomes between groups (Table 3). Conversely, long-term postoperative outcomes clearly differed among the groups. The high group was linked to a higher incidence of early recurrence after surgery (58.3%, *p* < 0.001). Moreover, the high group revealed a higher incidence of postoperative recurrence. Hepatic recurrence, especially, was a distinctive recurrence pattern in the high group (45.8%, *p* = 0.075). This result was similar to a previous large multicenter study [17]. The RFS and DSS curves for patients classified by preoperative CA19-9 values are shown in Fig. 1A, B. The RFS time was significantly shorter in the high group than in others (median survival time, 5.5 months, *p* < 0.001). The DSS was also significantly shorter in the high group (median survival time, 19.4 months, *p* = 0.025). Taken together, these results, as well as past reports [4], indicate that CA19-9 is a strong prognostic predictor. These results supported the premise of the current study that PDAC with high CA19-9 levels has an extremely poor prognosis. In short, these cases are suitable for further analysis to achieve our objectives.

**Table 3:** Postoperative outcomes in the entire cohort

	All (*n* = 128)	Normal (*n* = 46)	Low (*n* = 24)	Intermediate (*n* = 34)	High (*n* = 24)	*p* value
Postoperative complications (Clavien–Dindo classification grade ≥ 3), *n*	24 (18.8)	6 (13.0)	4 (16.7)	8 (23.5)	6 (25.0)	0.537
Pancreatic fistula (ISGPF grade ≥ B), *n*	23 (18.0)	4 (8.7)	4 (16.7)	11 (32.4)	4 (16.7)	0.057
Postoperative hospital stay, day	19 (6–73)	19 (9–61)	18 (7–64)	22 (8–57)	22 (6–73)	0.531
Adjuvant chemotherapy, *n*	103 (83.1)	34 (79.1)	21 (87.5)	31 (91.2)	17 (73.9)	0.286
Recurrence within 6 months, *n*	36 (28.1)	5 (10.9)	3 (12.5)	14 (41.2)	14 (58.3)	< 0.001
Recurrence, *n*	98 (76.6)	30 (65.2)	18 (75.0)	28 (82.4)	22 (91.7)	0.071
Pattern of recurrence						
Loco-regional recurrence, *n*	54 (42.2)	20 (43.5)	11 (45.8)	14 (41.2)	9 (37.5)	0.942
Hepatic recurrence, *n*	37 (28.9)	10 (21.7)	4 (16.7)	12 (35.3)	11(45.8)	0.075
Peritoneal recurrence, *n*	19 (14.8)	9 (19.6)	1 (4.2)	4 (11.8)	5 (20.8)	0.271
Other distant recurrence, *n*	21 (16.4)	2 (4.3)	7 (29.2)	7 (20.6)	3 (12.5)	0.031

*ISGPF* the International Study Group of Pancreatic Fistula, *ISGPS* the International Study Group of Pancreatic Surgery

### Preparation of dataset for proteomic profiling

To gain comprehensive insights into the characterization of PDAC with a high CA19-9 level, we analyzed surgically resected PDAC tissues using a proteomic approach. To clearly uncover distinctive differences between the groups, we excluded the intermediate groups from the target in proteomics analysis. Furthermore, we excluded the normal group, which might include the patients with the Lewis blood group-negative phenotype [Le(a-b-)], to exclude heterogeneity due to the Lewis antigen [4, 18]. Finally, 15 PDAC tissues chosen from the low and high groups, and 33 non-tumor parts from all the groups were analyzed. The comparison of clinical characteristics, intraoperative and postoperative outcomes, and pathological characteristics between the low and the high groups are presented in Supplemental Tables 1–3. Moreover, the comparisons of RFS and DSS curves between the low and the high groups are shown in Fig. 1C, D. These two groups clearly revealed a difference in the clinical findings and course, after they were determined suitable for further proteomic analysis to achieve our objectives.

### Proteomic profiling of the PDAC with high CA19-9 level

We identified 1060 quantified proteins to determine the significance of differences in protein expression by a *q* value cutoff set at < 0.1 as the threshold. Then, the protein profiles of the pancreas in the low, high, and non-tumor groups were compared. First, we performed two group comparisons to find the discriminating variables between the non-tumor group and the low or the high group, respectively. Comparing the non-tumor and the low group, 358 of 1060 proteins (33.8%) were identified as having differentially expressed proteins after statistics using t test with Benjamini–Hochberg correction (*p* = 0.050, *q* = 0.145). The biological replicates were studied using principal component analysis (Supplemental Fig. 1A). Among them, 239 proteins (66.8%) were up-regulated and 119 proteins (33.2%) were down-regulated in the low group (Supplemental Fig. 1B).

In a comparison between the non-tumor and the high group, 373 of 1060 proteins (35.2%) were significantly altered after the statistics were adjusted (*p* = 0.050, *q* = 0.139). A principal component analysis demonstrating the biological replicates is shown in Supplemental Fig. 1C. The number of differentially expressed proteins in the high group was relatively similar to those in the low group. Among them, 249 proteins (66.8%) were up-regulated and 124 proteins (33.2%) were down-regulated in the high group (Supplemental Fig. 1D). In short, we discovered no significant proteomic differences in number between the low and the high group. Compared to the non-tumor group, most, but not all of these discriminating proteins, were up-regulated proteins.

### Distinctive altered protein in the PDAC with high CA19-9 level is associated with glycolysis

**Fig. 2:**
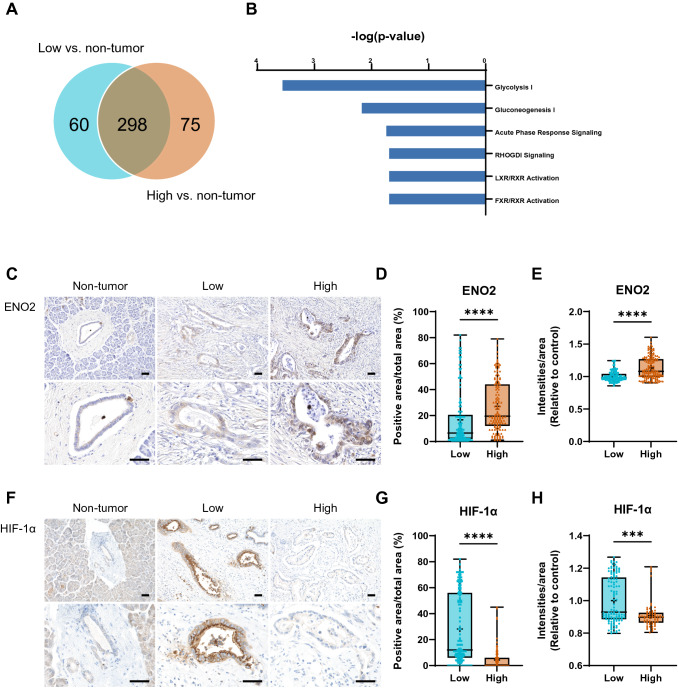
Distinctive altered protein in the PDAC with high CA19-9 level. **A** Venn diagrams showing unique and overlapping proteins among the comparisons. **B** Canonical pathway analysis using the 75 proteins exclusive to the high group. The top enriched categories of canonical pathways with a *p* value cutoff set at < 0.05 by Benjamini–Hochberg correction (− log (B–H *p* value) greater than 1.5). Glycolysis is significantly active in the PDAC with high CA19-9. **C** Representative immunohistochemistry images of ENO2 of resected PDAC specimens. The high CA19-9 group showed a remarkable increase in the number of ENO2+ cells. Scale bar = 50 μm. **D** ENO2+ area and duct area were calculated, and the percentages of ENO2+ area per duct area are plotted. **E** The intensities of ENO2+ signals and ENO2+ area were calculated, and the intensities of ENO2+ per ENO2+ area are expressed as arbitrary units (a.u.). **F** Representative immunohistochemistry images of HIF-1α of resected PDAC specimens. The high CA19-9 group showed a weak HIF-1α expression. Scale bar = 50 μm. **G** HIF-1α+ area and duct area were calculated, and the percentages of HIF-1α+ area per duct area are plotted. **H** The intensities of HIF-1α+ signals and HIF-1α+ area were calculated, and the intensities of HIF-1α+ per HIF-1α+ area are expressed as arbitrary units (a.u.). Statistical significance was determined using the Mann–Whitney *U* test. ***p < 0.001, ****p < 0.0001

We then determined the significant specific expressed proteins that appeared exclusively in one particular cohort and neither of the other cohorts. We generated a Venn diagram. We identified 60 of 358 proteins in the low group differentially expressed from the non-tumor group, and 75 of 373 proteins in the high group different from the non-tumor group, which were exclusive to each particular cohort (Fig. 2A). We further performed QIAGEN Ingenuity Pathway Analysis (QIAGEN IPA, QIAGEN Inc., Valencia, CA, USA) canonical pathway analysis using the 75 exclusive proteins in the high group. Figure 2B shows the top enriched categories of canonical pathways with a *p* value cutoff set at < 0.05 by Benjamini–Hochberg correction (− log (B–H *p* value) greater than 1.5). As a result, glycolysis was the most significantly enriched pathway. These data speculated differences in the contribution of glycolysis between the low and high groups.

### Glycolysis is significantly active in PDAC with a high CA19-9 level

Enolase 2 (ENO2) is a key glycolytic enzyme in the metabolic process of glycolysis, which is associated with worsened prognosis in various cancer [19, 20]. To clarify the possibility that glycolysis is activated in high CA19-9 PDAC, we next assessed the protein expression of ENO2 in the resected pancreas (Fig. 2C). Both the non-tumor group and the low group showed a weak expression in the duct structure, while the cytoplasm of adenocarcinoma and stromal tissues in the high group showed a strong ENO2 expression. In contrast to the low group, the high group showed a significant threefold increase in the expression of ENO2 in ductal adenocarcinoma formation (*p* < 0.001) (Fig. 2D, E), suggesting glycolysis has a crucial role in high CA19-9 PDAC.

Next, we sought to find the trigger promoting glycolysis. Glycolysis increases under hypoxic conditions [21, 22]. To investigate the involvement of hypoxia, we performed IHC staining for hypoxia-inducible factor 1α (HIF-1α), which is induced under hypoxic conditions (Fig. 2F). As a result, HIF-1α expression was found in the cytoplasm of adenocarcinoma and stroma in the low group. However, the high group showed a weaker HIF-1α expression than the low group (*p* < 0.001) (Fig. 2G, H). These results suggest that the promotion of glycolysis observed in the high group is not dependent on the hypoxic response.

### Differences in canonical pathways exist between the low and the high groups

To systematically determine an overview of the pathways changed in PDAC, the dataset that includes all the identified differentially expressed proteins was evaluated against the non-tumor group using IPA canonical pathway analysis. As a result, we discovered 92 enriched canonical pathways with a *p* value cutoff set at < 0.05, adjusted by Benjamini–Hochberg correction [− log (B–H *p* value)], greater than 1.5 in the low group. The top 30 pathways are shown in Supplemental Table 4. In the low group, SPINK1 Pancreatic Cancer Pathway was the most significantly activated pathway (*z* score: 1.155, *p* value: 3.82E−8, ratio: 0.2). The top five pathways according to *p* value were SPINK1 Pancreatic Cancer Pathway, BAG2 Signaling Pathway (*p* = 9.20E−8), Aryl Hydrocarbon Receptor Signaling (*p* = 4.22E−7), Clathrin-mediated Endocytosis Signaling (*p* = 7.81E−7), and Protein Ubiquitination Pathway (*p* = 8.22E−7).

Next, we discovered 82 enriched canonical pathways with a *p* value cutoff that was set at < 0.05, adjusted by Benjamini–Hochberg correction [− log (B–H *p* value)], greater than 1.5 in the high group. The top 30 pathways are shown in Supplemental Table 5. Even in comparing the non-tumor and the high group, SPINK1 Pancreatic Cancer Pathway was the most significantly activated pathway (*z* score: 1.732, *p* value: 6.41E−8, ratio: 0.2). The top five pathways according to *p* value were SPINK1 Pancreatic Cancer Pathway, EIF2 Signaling (*p* = 1.05E−7), Aryl Hydrocarbon Receptor Signaling (*p* = 1.05E−7), Clathrin-mediated Endocytosis Signaling (*p* = 2.3E−7), and Actin Cytoskeleton Signaling (*p* = 2.44E−7).

**Fig. 3:**
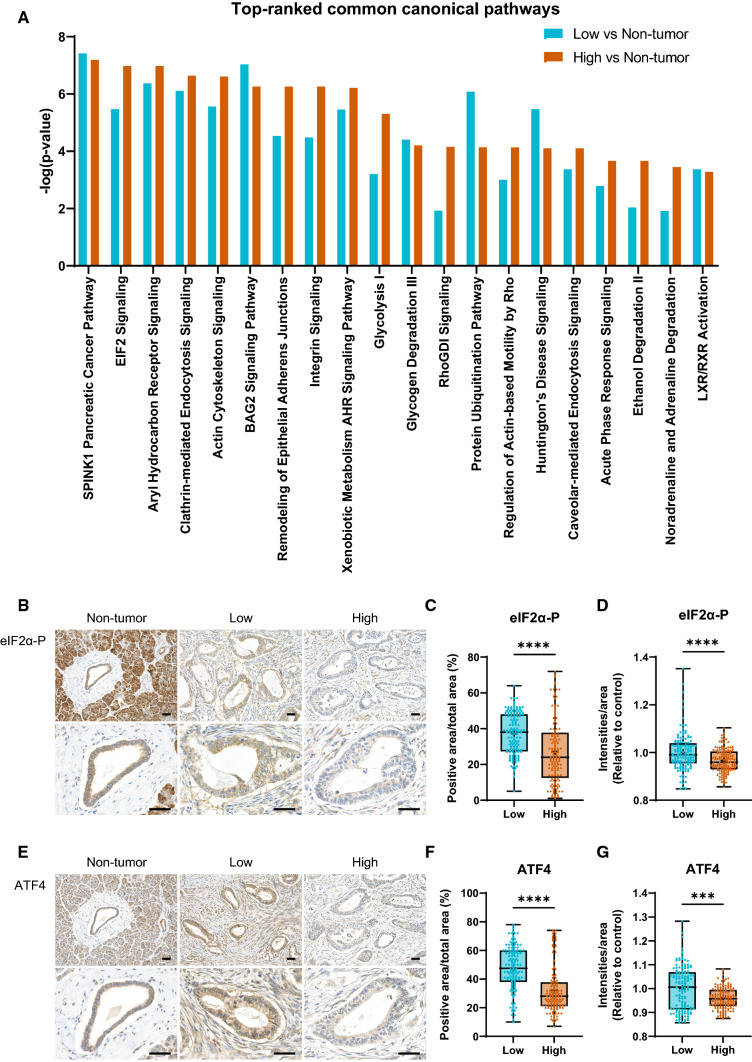
**A** Top-ranked common canonical pathways of the low and the high groups. The left *y*-axis represents the − log (*p* value). EIF2 signaling is different between the low and high groups. **B** Representative immunohistochemistry images of the eIF2α-P of resected PDAC specimens. The high CA19-9 group showed significant decreases in the expression of eIF2α-P. Scale bar = 50 μm. **C** Positive eIF2α-P area and duct area were calculated, and the percentages of eIF2α-P+ area per duct area are plotted. **D** The intensities of eIF2α-P signals and eIF2α-P area were calculated, and the intensities of eIF2α-P+ per eIF2α-P+ area are expressed as arbitrary units (a.u.). **E** Representative immunohistochemistry images of ATF4 of resected PDAC specimens. The high CA19-9 group showed a weak ATF4 expression. Scale bar = 50 μm. **F** ATF4+ area and duct area were calculated, and the percentages of ATF4+ area per duct area are plotted. **G** The intensities of ATF4+ signals and ATF4+ area were calculated, and the intensities of ATF4+ per ATF4+ area are expressed as arbitrary units (a.u.). Statistical significance was determined using the Mann–Whitney *U* test. ***p < 0.001, ****p < 0.0001

Figure 3A shows a side-by-side comparison of the results of the canonical pathway analyses. Consequently, we discovered cancer evolution-related pathways, including cellular growth or cell migration, were more enriched in the high group. Interestingly, in addition to glycolysis, various pathways involved in metabolism such as protein synthesis (EIF2 signaling) and lipid metabolism (Xenobiotic Metabolism AHR Signaling Pathway) were more notable in the high group.

### Proteostasis is different between the low and the high groups

EIF2 signaling plays an essential role in protein homeostasis (proteostasis). Phosphorylation of the α subunit of eukaryotic initiation factor 2 (eIF2α) reduces general translation initiation, leading to negative regulation of protein synthesis [23–25]. To clarify how EIF2 signaling differs between the groups, we next assessed the protein expression of phosphorylated eIF2α (eIF2α-P) in the resected pancreas (Fig. 3B). As a result, the non-tumor group demonstrated the strongest expression across the groups. In contrast, the low group significantly decreased to four-fifths of the non-tumor group. Furthermore, the high group significantly decreased to half of the non-tumor group. The high group showed significant decreases in the expression of eIF2α-P (*p* < 0.001) compared to the low groups (Fig. 3C, D), suggesting no decrease of protein synthesis in high CA19-9 PDAC.

When eIF2α is phosphorylated, general translation is stopped, but only translation of the transcription factor of activating transcription factor 4 (ATF4) is promoted, leading to increases in ATF4 protein expression [25–27]. We then assessed the protein expression of ATF4 in the same resected pancreas (Fig. 3E). Similar to eIF2α-P staining, the expression in the high group significantly decreased to half of the other groups (*p* < 0.001) (Fig. 3F, G). ATF4 can control amino acid metabolism, by promoting the transcription of amino acid metabolism-related genes [27–29]. Collectively, these data suggested that the difference in ATF4 expression between the low and the high groups can reflect the difference in proteostasis as well as amino acid metabolism.

### Metabolic reprogramming behind the malignant phenotype in the PDAC with high CA19-9 level

**Fig. 4:**
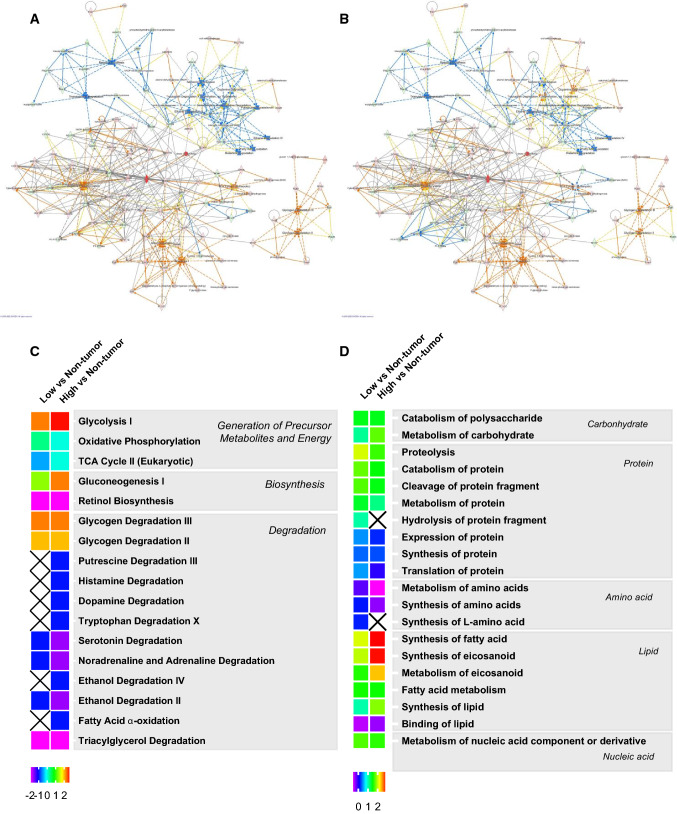
Interactions of significantly enriched metabolic-related canonical pathways with their associated proteins. Green and red protein nodes represent decreased and increased levels, respectively, compared to non-tumor parts. The deeper the color, the stronger the activity. Nodes of canonical pathways in blue indicate predicted inhibition, while those in orange indicate predicted activation. The deeper the color, the more confident the prediction. Lines of interaction show predicted relationships. Orange lines are leading to activation, while blue lines are leading to inhibition based on the findings. Yellow lines indicate that the direction of the findings does not match the direction of the expression variation in the dataset. Lines in gray indicate that an effect is not predicted. **A** Interactions based on the data from a comparison between the non-tumor and the low group. **B** Interactions based on the data from a comparison between the non-tumor and the high group. Heatmap comparing IPA functional analyses of the two comparisons. The heatmap shows the functional classifications of identified metabolism-related canonical pathways and biological functions. IPA functional analysis was performed using significantly differentially expressed proteins from the non-tumor group in the low and high group, respectively. **C** Canonical pathways and their categories. **D** Biological functions. This heatmap was drafted according to *z* score values, where higher *z* scores, in red, indicate activation, while lower *z* scores, in blue, indicate inhibition

Based on the above results, such as differences in carbohydrate and protein metabolism, we further analyzed the altered metabolic pathways in the high group. IPA was also used to gain a global perspective on the significantly enriched metabolic pathways in the low and the high groups (Fig. 4A, B). A heatmap containing some representative classifications of metabolism-related canonical pathways and biological functions is shown in Fig. 4C, D. These figures show an organized set of those metabolic pathways by small category. As a result, we found a heterogeneity of various metabolic processes, including carbohydrate, protein, amino acid, lipid, and nucleic acid between the groups. The regulation of various metabolic processes in cancer is called metabolic reprogramming [10, 30]. In other words, metabolic reprogramming in the high group was different from that of the low group. Collectively, these data suggest that differences in metabolic reprogramming can yield a difference in the malignant phenotype of the PDAC.

## Discussion

We have characterized the metabolic landscape of PDAC comprehensively by proteomics analysis using clinically resected pancreases. Our study demonstrated the differences in the metabolic state between low and high CA19-9 PDAC patients. Furthermore, our study also indicated that this heterogeneity of metabolic adaptation was involved with further cancer evolution and poor prognosis. In other words, an enhanced metabolic adaptive capacity is closely related to aggressive malignant phenotypes. Our results suggest that CA19-9 can help estimate the metabolic adaptive capacity for energy supply to PDAC.

PDAC cases are metabolically different from the normal pancreas, in a manner that reflects the hostile tumor microenvironment, including poor nutrition, hypoxia, acidosis, and high interstitial pressure [30]. Thus, PDAC needs to adapt its metabolism to the hostile conditions to survive and proliferate. Several lines of evidence have shown that these metabolic adaptations also make PDAC cells more motile, invasive, and chemotherapy/radiotherapy resistant. Therefore, metabolic reprogramming is a novel area of interest in PDAC therapeutic strategy [30]. Our results indicated that there are differences in metabolic adaptation even within PDAC patients and that these differences cannot be ignored when establishing therapeutic strategies for PDAC.

Metformin, an anti-diabetes medicine, is one of the promising candidates directly targeting PDAC metabolism. Indeed, this is now in clinical trials [30]. We therefore simulated the impact of metformin in our dataset (Supplemental Fig. 2). Consequently, we found different predicted phenotypes between the low and high CA19-9 groups. These simulations indicate that among PDAC patients, some cases may benefit from metformin, and others may not. In short, it suggests that the heterogeneity of metabolic status, as our study showed, should be taken into account to achieve the desired results of the clinical trials. As described in an excellent review by Andersen et al., PDAC metabolism targeting treatment will require personalized protocols [30].

Most findings surrounding metabolic adaptation have been obtained using cell line-based and animal model-based analyses. However, metabolic adaptation is responsible for microenvironmental conditions which include various components. Thus, it is important to be cautious about the conclusions drawn from homogeneous cell populations. In short, cancer metabolism demonstrates different characteristics in laboratory cell culture settings than in vivo [31]. Consequently, most observations of human cancer metabolic reprogramming in vivo have been conducted using bulk tumor analysis similar to ours. The metabolic landscape based on a comprehensive analysis of surgically resected human tissue is one of the strengths of this study. In contrast, bulk measurements tend to mask differences between cells by averaging expression levels. To reach the heterogeneity of metabolic adaptation in each cancer cell, a single-cell analysis would be better [31]. However, preparing cells for single-cell analysis from fresh surgical human specimens may interfere with the pathological evaluation of PDAC, especially in a tiny tumor. Thus, in real clinical practice, it is ethically challenging to perform single-cell analysis in a large number of PDAC cases.

Recently, a dissociation between technically resectable and biologically resectable cases has been recognized. Based on this concept, some high CA19-9 PDAC patients would be considered to have biologically borderline or unresectable PDAC even if the patient’s condition is judged as resectable PDAC based on image-based resectability criteria. Indeed, there were high CA19-9 PDAC cases in the present cohort for whom performing up-front surgery led to unsatisfactory outcomes. In short, among high CA19-9 PDAC, there is a diversity of imaging findings, from those that clearly show distant metastases to those that are considered resectable. Our findings were obtained from resected PDAC tissue from up-front surgery, not from patients who received neoadjuvant therapy prior to surgery. Though this is one of the limitations of this study, the samples of high CA19-9 PDAC used in this study might not be representative of PDAC with high CA19-9 in real clinical situations.

In the future a prospective, proteomic analysis using a large dataset that additionally includes both a normal group excluding the Lewis antigen-negative cases and an intermediate group is needed. Showing metabolic heterogeneity with gradient changes among the groups would be more useful to get insight into metabolic targeted therapy for this lethal disease.

Though we demonstrated the differences in metabolism status among PDAC cases, we could not conclude which was the first adapted pathway. Moreover, it was unclear whether metabolic adaptation changes occur sequentially in a certain direction. Establishing a temporal hierarchy of these metabolic adaptations is challenging. However, metabolic pathways interact with each other, and all of them have the potential to become the ultimate trigger for cancer [32]. Thus, it is as important to identify whether these metabolic adaptations are reversible, as it is to identify temporal hierarchy. To gain a wider perspective, we need to clarify the plasticity of metabolic adaptation in PDAC.

In conclusion, our study indicated that there were differences in metabolic adaptation among PDAC cases, which are involved in cancer evolution as well as the prognosis. CA19-9 can help estimate the metabolic adaptive capacity for a full energy supply to PDAC evolution. Recognition of this individual heterogeneity of metabolic status will be essential for the success of clinical trials of metabolism targeting therapy for PDAC. Consequently, PDAC metabolism targeting treatment will require personalized protocols. We expect that future studies will expand our understanding of the plasticity of metabolic reprogramming in the natural history of PDAC.

## Supplementary Information

Below is the link to the electronic supplementary material.

Supplemental Figure 1 Principal component analysis and hierarchically clustered heatmap. (A, C) Principal component analysis (PCA). Gray dots are samples from the non-tumor groups, green dots are samples from the low groups and brown dots are samples from the high groups. Proteins passing the p-value cutoff set at < 0.05 for ANOVA with Benjamini–Hochberg correction were used for PCA. (B, D) A hierarchically clustered heatmap of the relative abundance of proteins in the two groups. The orange blocks represent the up-regulated proteins, and the blue blocks represent down-regulated proteins. Differentially expressed proteins were identified using the ttest with Benjamini–Hochberg correction, with a p-value cutoff set at < 0.05 and log2FC set at ≥ 1. (PPTX 4095 KB)

Supplemental Figure 2 The causal relationship between the metabolic-related canonical pathways and cancer evolution. (A) Interactions based on the data from a comparison between the non-tumor and the low group. (B) Interactions based on the data from a comparison between the non-tumor and the high group. Virtual simulation of metformin administration on the causal relationship generated by our dataset. (C) Predicted causality based on the data from a comparison between the non-tumor and the low group. (D) Predicted causality based on the data from a comparison between the non-tumor and the high group. Green and red protein nodes represent decreased and increased levels, respectively, compared to non-tumor parts. The deeper the color, the stronger the activity. Nodes of canonical pathways in blue indicate predicted inhibition, while those in orange indicate predicted activation. The deeper the color, the more confident the prediction. Lines of interaction show predicted relationships. Orange lines are leading to activation, while blue lines are leading to inhibition based on the findings. Yellow lines indicate that the direction of the findings does not match the direction of the expression variation in the dataset. Lines in gray indicate that an effect is not predicted. Nodes of metformin in red indicate that it is activated in silico. (PPTX 8368 KB)

Supplementary file3 (DOCX 26 KB)

Supplementary file4 (DOCX 58 KB)

## Data Availability

The proteomic data are available online using access number “PXD025975’’ for the Proteome Xchange site [33] and access number “JPST001172’’ for the jPOST Repository [34].

## Abbreviations

ATF4: Activating transcription factor 4
B–H: Benjamini–Hochberg correction
CA19-9: Carbohydrate antigen 19-9
DSS: Disease-specific survival
EIF2: Eukaryotic initiation factor 2
ENO2: Enolase 2
HIF-1α: Hypoxia-inducible factor 1α
IHC: Immunohistochemistry
LC–MS/MS: Liquid chromatography with tandem mass spectrometry
PDAC: Pancreatic ductal adenocarcinoma
RFS: Recurrence-free survival
