# Supplementary figures and images for: Heterogeneity of metabolic adaptive capacity affects the prognosis among pancreatic ductal adenocarcinomas
Source: J Gastroenterol. 2022 Jul 3;57(10):798–811. doi: 10.1007/s00535-022-01898-0 (PMC9522820; doi:10.1007/s00535-022-01898-0)

## Slide 1
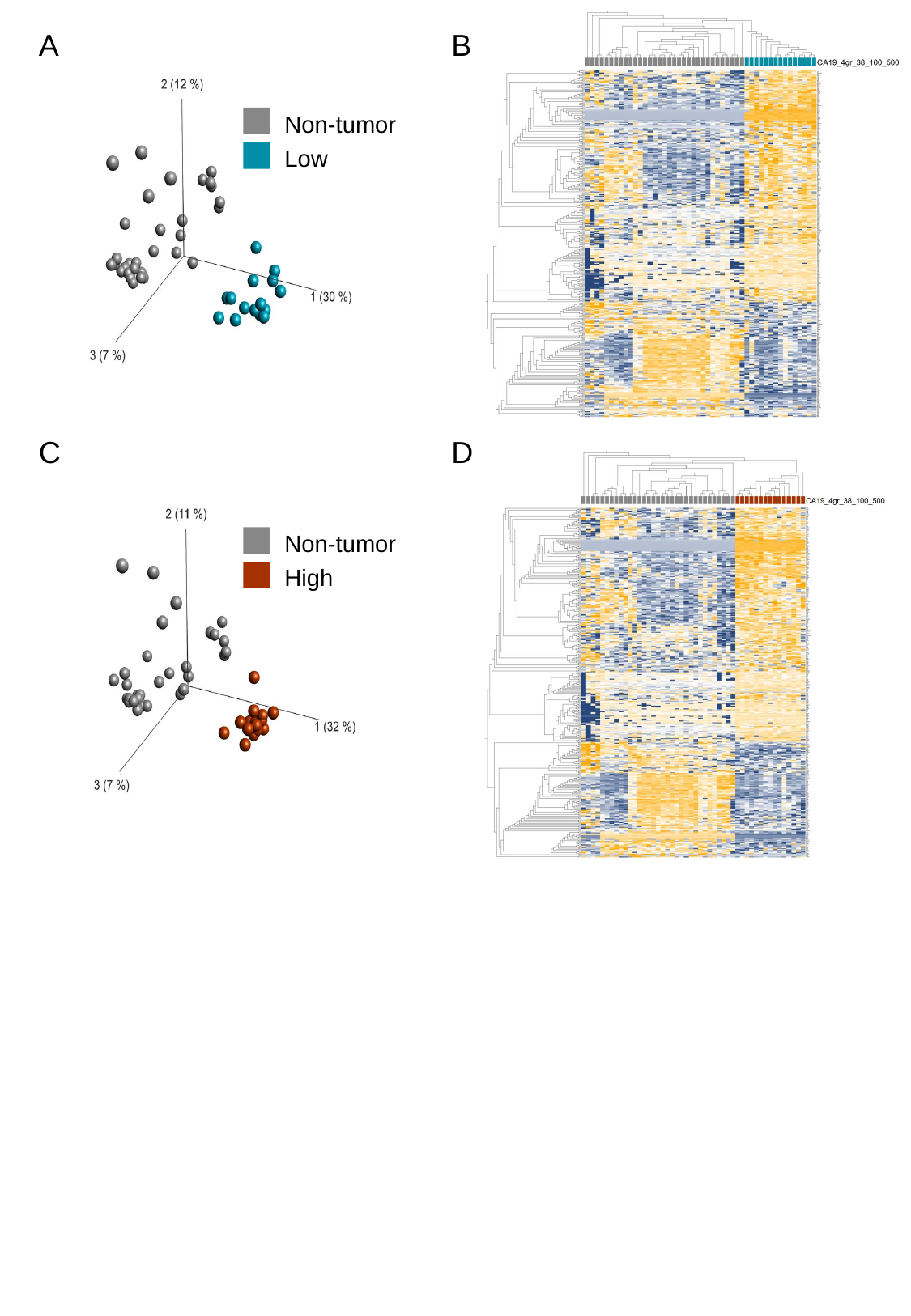

A
B
Non-tumor
Low
C
D
Non-tumor
High

Supplement: Supplementary file 1 — Supplemental Figure 1 Principal component analysis and hierarchically clustered heatmap. (A, C) Principal component analysis (PCA). Gray dots are samples from the non-tumor groups, green dots are samples from the low groups and brown dots are samples from the high groups. Proteins passing the p-value cutoff set at < 0.05 for ANOVA with Benjamini–Hochberg correction were used for PCA. (B, D) A hierarchically clustered heatmap of the relative abundance of proteins in the two groups. The orange blocks represent the up-regulated proteins, and the blue blocks represent down-regulated proteins. Differentially expressed proteins were identified using the ttest with Benjamini–Hochberg correction, with a p-value cutoff set at < 0.05 and log2FC set at ≥ 1. (PPTX 4095 KB) [file 535_2022_1898_MOESM1_ESM.pptx]
