# Supplementary material for: Heterogeneity of metabolic adaptive capacity affects the prognosis among pancreatic ductal adenocarcinomas
Source: J Gastroenterol. 2022 Jul 3;57(10):798–811. doi: 10.1007/s00535-022-01898-0 (PMC9522820; doi:10.1007/s00535-022-01898-0)
Supplement: Supplementary file 2 — Supplemental Figure 2 The causal relationship between the metabolic-related canonical pathways and cancer evolution. (A) Interactions based on the data from a comparison between the non-tumor and the low group. (B) Interactions based on the data from a comparison between the non-tumor and the high group. Virtual simulation of metformin administration on the causal relationship generated by our dataset. (C) Predicted causality based on the data from a comparison between the non-tumor and the low group. (D) Predicted causality based on the data from a comparison between the non-tumor and the high group. Green and red protein nodes represent decreased and increased levels, respectively, compared to non-tumor parts. The deeper the color, the stronger the activity. Nodes of canonical pathways in blue indicate predicted inhibition, while those in orange indicate predicted activation. The deeper the color, the more confident the prediction. Lines of interaction show predicted relationships. Orange lines are leading to activation, while blue lines are leading to inhibition based on the findings. Yellow lines indicate that the direction of the findings does not match the direction of the expression variation in the dataset. Lines in gray indicate that an effect is not predicted. Nodes of metformin in red indicate that it is activated in silico. (PPTX 8368 KB) [file 535_2022_1898_MOESM2_ESM.pptx]

## Slide 1
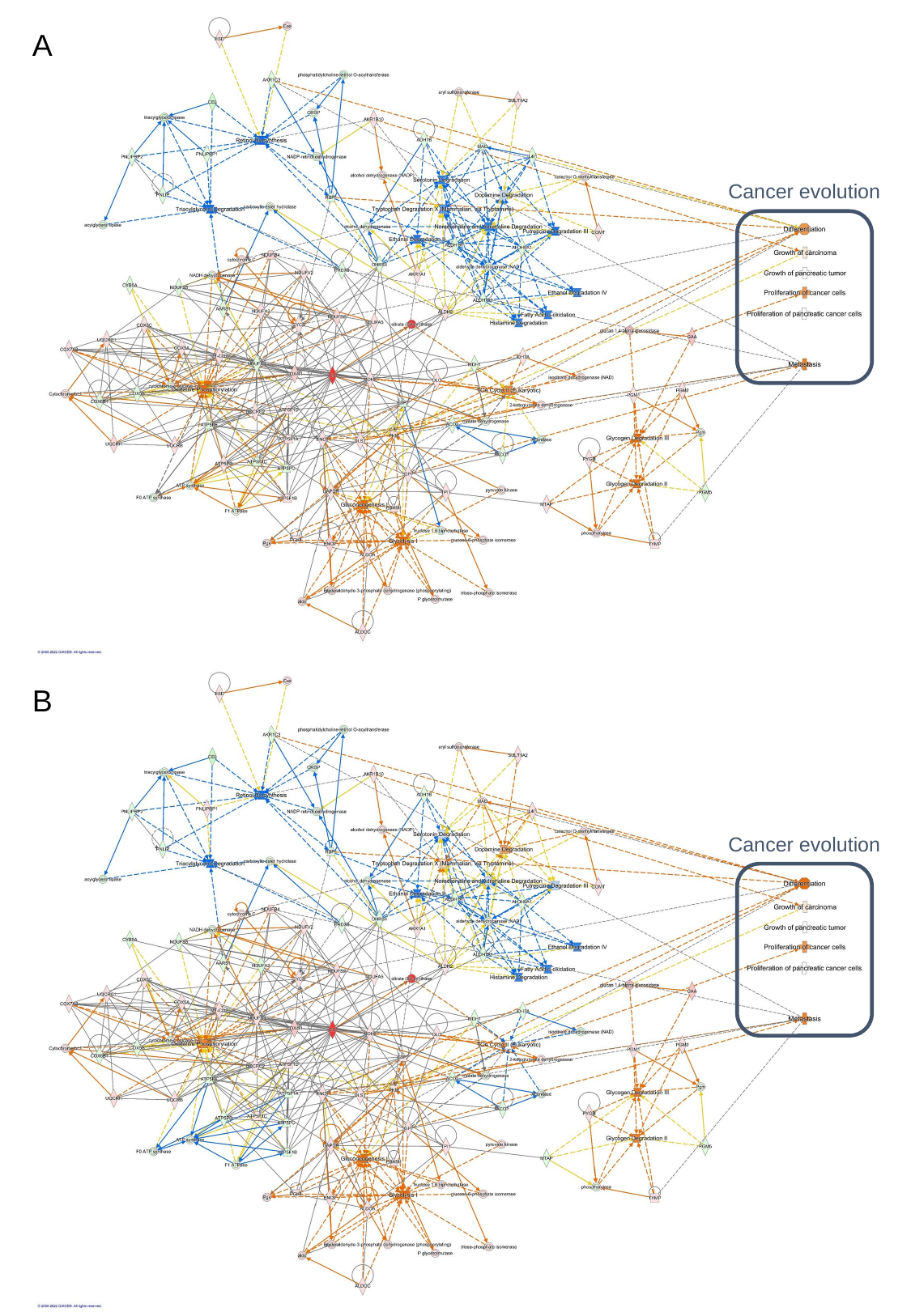

A
B
Cancer evolution
Cancer evolution

## Slide 2
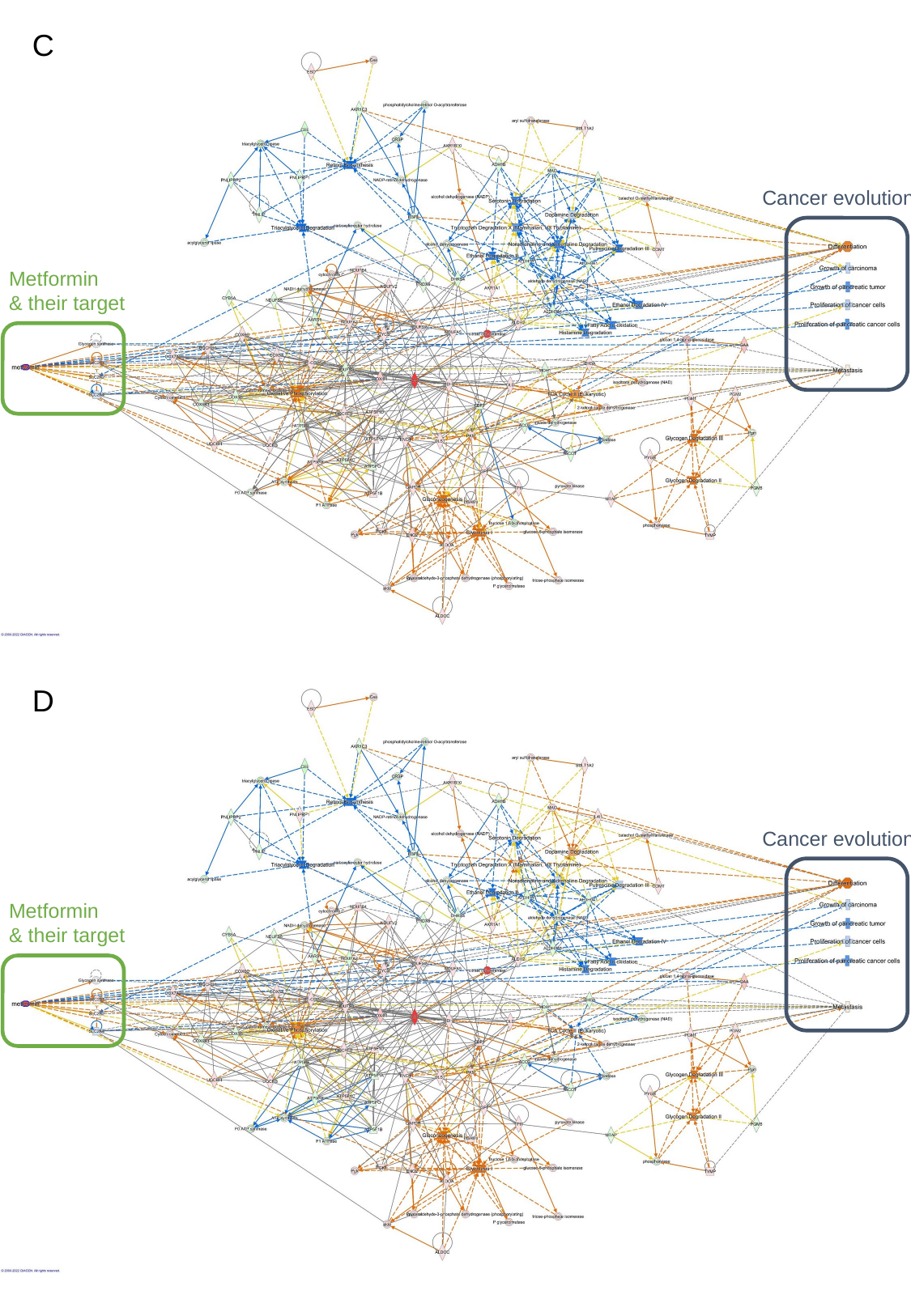

C
D
Cancer evolution
Metformin
& their target
Cancer evolution
Metformin
& their target
