# Supplementary material for: Heterogeneity of metabolic adaptive capacity affects the prognosis among pancreatic ductal adenocarcinomas
Source: J Gastroenterol. 2022 Jul 3;57(10):798–811. doi: 10.1007/s00535-022-01898-0 (PMC9522820; doi:10.1007/s00535-022-01898-0)
Supplement: Supplementary file 3 — Supplementary file3 (DOCX 26 KB) [file 535_2022_1898_MOESM3_ESM.docx]

**Supplemental Digital Content 1 (Supplemental Content 1)**

**METHODS**

*Tissue section preparation*

Approximately ten-microgram sections were cut from each of the formalin-fixed and paraffin-embedded PDAC blocks. Based on the findings on H&E staining, we selectively cut out sections from the cancerous parts. Additionally, we cut out the section from the non-tumor part, where the pancreatic parenchyma was farthest from the cancerous area in a surgically resected specimen. In order of newest first, we selected the blocks deemed to be in usable condition for MS analysis. Finally, 15 cancer tissues from the low group, 15 cancer tissues from the high group, and 33 non-tumor tissues were prepared for proteomic analysis for this study.

*Liquid chromatography with tandem mass spectrometry (LC-MS/MS)*

Formalin-fixed and paraffin-embedded pancreas tissues were deparaffinized using two changes of xylene and then washed with descending concentrations of ethanol. Proteins in the deparaffinized tissues were extracted in 20% (w/v) of 200 mM Tris-HCl (pH 8.8) containing 2% SDS and 0.2 M DTT, which was followed by incubation at 100˚C for 20 min and shaking incubation at 80˚C for 2 h. The extracted proteins were precipitated by adding acetone. The precipitated proteins were resuspended in 10 μl of 500 mM ammonium bicarbonate and denatured with an equivalent volume of trifluoroethanol. Free cysteine residues were alkylated with 4 μl of 200 mM iodoacetamide for 60 min at room temperature in the dark and the remaining iodoacetamide was quenched by adding 1 μl of 200 mM DTT. The samples were then mixed with 300 μl of 100 mM ammonium bicarbonate. Fifteen microliters of the sample was diluted with 85 μl of 100 mM ammonium bicarbonate and incubated with 1 μg trypsin (TPCK treated, AB Sciex, Framingham, MA, USA) at 37°C for 18 h. The samples were desalted with C18 ZipTip (Millipore, Bedford, MA, USA) and eluted with H2O/acetonitrile (5/5; v/v). The ZipTip eluates were dried in a vacuum centrifuge. Desalted samples were rehydrated in 0.1% formic acid (FA) and were analyzed by liquid chromatography mass spectrometry (LC-MS) using a nanoLC Eksigent 400 system (Eksigent, AB Sciex), coupled online to a TripleTOF6600 mass spectrometer (AB Sciex). Peptide separation was performed using liquid chromatography with a trap and elution conﬁguration using a nano trap column (350 μm × 0.5 mm, 3 μm, 120 Å, AB Sciex) and a nano ChromXP C18 reverse phase column (75 μm × 15 cm, 3 μm, 120 Å, AB Sciex) at 300 nl/min with a 90 min linear gradient of 8-30% acetonitrile in 0.1% FA, and then, with a 10 min linear gradient of 30% to 40% acetonitrile in 0.1% FA. The mass spectrometer was operated in information-dependent acquisition (IDA) mode, scanning full spectra (400–1500 m/z) for 250 ms, followed by up to 30 MS/MS scans (100–1800 m/z for 50 ms each), for a cycle time of 1.8 s. Candidate ions with a charge state between +2 and + 5 and counts above a minimum threshold of 125 counts per second were isolated for fragmentation, and one MS/MS spectrum was collected for 12 s before adding those ions to the exclusion list. Rolling collision energy was used with a collision energy spread of 15. The mass spectrometer was operated using the Analyst TF 1.7.1 software program (AB Sciex). For data dependent acquisition (DDA, SWATH acquisition), the parameters were set as follows: 100 ms TOF MS scan, followed by 200 variable SWATH windows, each at a 50 ms accumulation time, for m/z 400–1250. MS/MS SWATH scans, which were set at a 5 Da window overlapping by 1 Da for m/z 400–1250 and varied on each side of the mass range. The total cycle time was 9.6 s. A rolling collision energy (CE) parameter script was used to automatically control the CE.

*Proteomics data analysis*

Acquired spectra were searched against the UniProt reviewed database using the Paragon algorithm embedded in the ProteinPilot 5.0.1 software program (AB Sciex), with the following search parameters: (i) sample type: identiﬁcation, (ii) Cys alkylation: iodoacetamide, (iii) digestion: trypsin, (iv) instrument: TripleTOF 6600, (v) species: Homo sapiens, (vi) ID focus: biological modiﬁcations, (vii) detected protein threshold: > 0.05 (10% conﬁdence). The detected protein threshold was set to the minimum level to enhance the number of wrong answers to enable the curve ﬁtting by an independent FDR analysis.^1^ This was carried out by the target-decoy approach provided with the ProteinPilot software program, which was used to assess the quality of the identiﬁcations. Positive identiﬁcations were considered to be when identiﬁed proteins and peptides reached a 1% local FDR.^2^ The resulting group file was loaded into Peakview (v2.2.0, AB Sciex) and peaks from SWATH runs were extracted with a peptide confidence threshold of 99% and a false discovery rate < 1%. The SWATH ﬁles were then exported to the MarkerView software program (version 1.3.0.1; AB Sciex) and the peak areas of individual peptides were normalized to the sum of the peak areas of all detected peptides.

Proteomics data were visualized using Qlucore Omics Explorer (Qlucore, New York, NY, USA). Proteomic signatures were compared between the high-grade ne and low-grade ne groups. Differentially expressed proteins were identified using t-test with Benjamini-Hochberg correction, with a p-value cutoff set at < 0.05 and log2FC set at ≥1.

Furthermore, QIAGEN Ingenuity Pathway Analysis (QIAGEN IPA, QIAGEN Inc., Valencia, CA, USA) was performed to identify canonical pathways, protein interactions, and functional networks that are most significant to our proteomics results. The core analysis was carried out with the settings of indirect and direct relationships between molecules based on experimentally observed data, and data sources were considered from mammal databases in the Ingenuity Knowledge Base. Fisher’s exact test was used to determine the probability that biological functions and/or diseases were over-represented in the protein dataset.

*Immunohistochemistry (IHC)*

Immunohistochemical examination was performed on the deparaffinized section using the standard avidin- oxidase complex method with an automated immunostainer (Benchmark XT; Ventana Medbiotin-perical System, Tucson, AZ, USA). In brief, deparaffinized slides were treated with tris EDTA buffer (pH 7.8) at 95˚C for 44 min. The slides were treated with 5% non-fat dry milk at 37˚C for 15 min to block endogenous peroxides and protein. The slides were incubated with primary antibodies for 60 min at room temperature. The antibodies and dilution ratios were: anti neuron-specific enolase (ENO2) (1:200, M0873, Aglient DAKO, Santa Clara, CA, USA), anti-hypoxia inducible factor 1α (HIF-1α) (1:100, MAB5382, Millipore, Billerica, MA, USA), anti-Phosphorylated Eukaryotic initiation factor 2 (eIF2α-P) (1:100, Ab32157, Abcam, MA, USA), and anti-activating transcription factor 4 (ATF4) (1:200, 10835-1-AP, Proteintech Group, Chicago, IL, USA).

Pictures were obtained with an all-in-one microscope (BZ-X700, Keyence) and analyzed with BZ-X Analyzer (Keyence) software. Each ductal carcinoma formation was manually segmented with the software. Twenty carcinoma ducts were randomly selected in each slide to perform quantitative analysis of the positive cells. Positive areas and signaling intensities for the indicated antibodies and respective total areas were automatically calculated using Hybrid Cell Count (Keyence).

**References**

1. Tang WH, Shilov IV, Seymour SL. Nonlinear fitting method for determining local false discovery rates from decoy database searches. *J Proteome Res* 2008;7:3661-7.

2. Sennels L, Bukowski-Wills JC, Rappsilber J. Improved results in proteomics by use of local and peptide-class specific false discovery rates. *BMC Bioinformatics* 2009;10:179.
