# Supplementary material for: Heterogeneity of metabolic adaptive capacity affects the prognosis among pancreatic ductal adenocarcinomas
Source: J Gastroenterol. 2022 Jul 3;57(10):798–811. doi: 10.1007/s00535-022-01898-0 (PMC9522820; doi:10.1007/s00535-022-01898-0)
Supplement: Supplementary file 4 — Supplementary file4 (DOCX 58 KB) [file 535_2022_1898_MOESM4_ESM.docx]

**SUPPLEMENTAL TABLE 1: Comparison of the clinical characteristics and operation-related factors between the low and the high groups.**

|  | All  (n = 128) | Low (n = 24) | High  (n = 24) | P value |
| --- | --- | --- | --- | --- |
| Gender, male, n | 65 (50.8) | 11 (45.8) | 15 (62.5) | 0.247 |
| Age, year | 70 (50-85) | 72 (54-79) | 69 (52-82) | 0.347 |
| Body weight, kg | 56.0 (34.0-85.0) | 55.5 (38.5-76.6) | 56.9 (34.7-82.5) | 0.710 |
| Body mass index, kg/m^2^ | 22.3 (14.1-36.3) | 22.7 (16.2-28.3) | 22.3 (15.8-33.3) | 0.812 |
| Obstructive jaundice, n | 60 (46.9) | 10 (41.7) | 12 (50.0) | 0.562 |
| Diabetes mellitus, n | 43 (33.6) | 12 (50.0) | 8 (33.3) | 0.242 |
| *Laboratory values* |  |  |  |  |
| WBC, /µL | 5165 (2230-11020) | 5490 (2230-7460) | 5200 (3980-8680) | 0.877 |
| Hemoglobin, g/dL | 12.7 (7.2-16.3) | 12.6 (9.9-14.9) | 12.1 (10.4-14.7) | 0.451 |
| Hematocrit, % | 37.2 (22.8-46.1) | 37.3 (29.3-43.0) | 36.3 (29.6-43.5) | 0.650 |
| Platelets, ×10^3^/µL | 222 (64-513) | 192 (96-393) | 242 (64-358) | 0.132 |
| CRP, mg/dL | 0.14 (0.02-9.59) | 0.11 (0.02-7.30) | 0.29 (0.02-5.40) | 0.152 |
| Albumin, g/dL | 3.9 (2.0-5.7) | 3.9 (2.4-5.7) | 3.9 (2.5-4.6) | 0.780 |
| Total protein, g/dL | 6.8 (4.9-8.9) | 6.6 (5.4-8.9) | 6.8 (5.3-8.1) | 0.909 |
| Creatinine, mg/dL | 0.64 (0.40-1.43) | 0.65 (0.46-1.10) | 0.60 (0.43-1.30) | 0.796 |
| AST, U/L | 29 (11-406) | 27 (13-91) | 52 (15-287) | 0.055 |
| ALT, U/L | 35 (9-627) | 28 (12-210) | 73 (12-443) | 0.043 |
| GTP, U/L | 65 (9-1720) | 83 (16-529) | 104 (9-893) | 0.392 |
| Total bilirubin, mg/dL | 0.8 (0.2-32.7) | 0.8 (0.3-24.0) | 1.7 (0.2-24.1) | 0.157 |
| Amylase, U/L | 74 (17-737) | 82 (35-462) | 78 (17-231) | 0.741 |
| Total cholesterol | 187 (84-653) | 173 (129-258) | 197 (84-563) | 0.225 |
| HbA1c | 6.0 (4.4-11.9) | 6.4 (4.8-9.8) | 6.8 (4.4-11.9) | 0.946 |
| CA19-9, U/mL | 71 (1-9675) | 61 (38-96) | 1401 (511-9675) | < 0.001 |
| CEA, ng/mL | 2.7 (0.5-37.0) | 2.8 (0.7-37.0) | 4.3 (1.0-17.0) | 0.029 |
| DUPAN, U/mL | 96 (22-16000) | 48 (22-625) | 523 (31-10800) | < 0.001 |
| SPAN, U/mL | 51 (2-2284) | 38 (24-142) | 496 (153-1667) | < 0.001 |
| *Operative outcomes* |  |  |  |  |
| Procedure, n |  |  |  | 0.122 |
| Pancreaticoduodenectomy | 83 (64.8) | 12 (50.0) | 18 (75.0) |  |
| Distal pancreatectomy | 40 (31.3) | 10 (41.7) | 6 (25.0) |  |
| Total pancreatectomy | 5 (3.9) | 2 (8.3) | 0 |  |
| Operation time, min | 310 (91-647) | 294 (139-573) | 310 (130-513) | 0.427 |
| Intraoperative blood loss, mL | 765 (90-3915) | 775 (155-2270) | 1003 (200-3915) | 0.115 |
| Intraoperative ABT | 24 (18.8) | 5 (20.8) | 7 (29.2) | 0.505 |
| Portal vein resection, n | 19 (14.8) | 2 (8.3) | 5 (20.8) | 0.416 |
| Postoperative CA19-9, U/mL | 18 (1-1065) | 16 (6-33) | 147.5 (9-1065) | < 0.001 |
| CA19-9 elevation after surgery, n | 6 (4.7) | 0 | 0 | n/a |

ABT, allogeneic red blood cell transfusion; CA19-9, carbohydrate antigen 19-9; CEA, carcinoembryonic antigen; DUPAN, duke pancreatic monoclonal antigen; n/a, not applicable; SPAN, s-pancreas antigen.

**SUPPLEMENTAL TABLE 2: Comparison of pathological characteristics between the low and the high groups.**

|  | All  (n = 128) | Low (n = 24) | High  (n = 24) | P value |
| --- | --- | --- | --- | --- |
| Tumor size, mm | 30 (7-150) | 30 (11-150) | 39 (25-70) | 0.028 |
| UICC 8^th^ edition |  |  |  |  |
| T category, n |  |  |  | 0.220 |
| T1 | 16 (12.5) | 2 (8.3) | 0 |  |
| T2 | 79 (61.7) | 15 (62.5) | 13 (54.2) |  |
| T3 | 33 (25.8) | 7 (29.2) | 11 (45.8) |  |
| T4 | 0 | 0 | 0 |  |
| N category, n |  |  |  | 0.097 |
| N0 | 49 (38.3) | 13 (54.2) | 6 (25.0) |  |
| N1 | 49 (38.3) | 6 (25.0) | 12 (50.0) |  |
| N2 | 30 (23.4) | 5 (20.8) | 6 (25.0) |  |
| M category, n |  |  |  | > 0.999 |
| M0 | 117 (91.4) | 22 (91.7) | 23 (95.8) |  |
| M1^a^ | 11 (8.6) | 2 (8.3) | 1 (4.2) |  |
| UICC Stage, n |  |  |  | 0.216 |
| ⅠA | 12 (9.4) | 2 (8.3) | 0 |  |
| ⅠB | 24 (18.8) | 7 (29.2) | 3 (12.5) |  |
| ⅡA | 12 (9.4) | 4 (16.7) | 3 (12.5) |  |
| ⅡB | 45 (35.2) | 5 (20.8) | 12 (50.0) |  |
| Ⅲ | 24 (18.8) | 4 (16.7) | 5 (20.8) |  |
| Ⅳ | 11 (8.6) | 2 (8.3) | 1 (4.2) |  |
| R0 resection, n | 114 (89.1) | 24 (100.0) | 21 (87.5) | 0.234 |
| *Local invasion factor* |  |  |  |  |
| Bile duct invasion | 60 (46.9) | 10 (41.7) | 12 (50.0) | 0.562 |
| Duodenal invasion | 56 (43.8) | 8 (33.3) | 17 (70.8) | 0.009 |
| Serosal side of the anterior pancreatic tissue invasion | 28 (21.9) | 4 (16.7) | 5 (20.8) | > 0.999 |
| Retropancreatic tissue invasion | 103 (80.5) | 18 (75.0) | 21 (87.5) | 0.461 |
| Portal venous system invasion | 28 (21.9) | 5 (20.8) | 4 (16.7) | > 0.999 |
| Arterial system invasion | 20 (15.6) | 4 (16.7) | 3 (12.5) | > 0.999 |
| Extrapancreatic nerve plexus invasion | 28 (21.9) | 6 (25.0) | 5 (20.8) | 0.731 |
| Invasion of other organs | 6 (4.7) | 1 (4.2) | 2 (8.3) | > 0.999 |
| *Assessment of TME* |  |  |  |  |
| Lymphatic invasion |  |  |  | 0.403 |
| No evidence of invasion | 5 (3.9) | 1 (4.2) | 0 |  |
| Slight invasion | 24 (18.8) | 5 (20.8) | 2 (8.3) |  |
| Moderate invasion | 59 (46.1) | 12 (50.0) | 13 (54.2) |  |
| Marked invasion | 40 (31.3) | 6 (25.0) | 9 (37.5) |  |
| Venous invasion |  |  |  | 0.140 |
| No evidence of invasion | 7 (5.5) | 2 (8.3) | 0 |  |
| Slight invasion | 33 (25.8) | 5 (20.8) | 4 (16.7) |  |
| Moderate invasion | 58 (45.3) | 14 (58.3) | 11 (45.8) |  |
| Marked invasion | 30 (23.4) | 3 (12.5) | 9 (37.5) |  |
| Nerve invasion |  |  |  | 0.179 |
| No evidence of invasion | 5 (3.9) | 1 (4.2) | 0 |  |
| Slight invasion | 15 (11.7) | 5 (20.8) | 1 (4.2) |  |
| Moderate invasion | 34 (26.6) | 5 (20.8) | 4 (16.7) |  |
| Marked invasion | 74 (57.8) | 13 (54.2) | 19 (79.2) |  |
| Cancer-stroma relationship |  |  |  | 0.336 |
| Medullary type | 4 (3.1) | 2 (8.3) | 0 |  |
| Intermediate type | 74 (58.3) | 12 (50.0) | 12 (50.0) |  |
| Scirrhous type | 49 (38.6) | 10 (41.7) | 12 (50.0) |  |

TME, tumor microenvironment; UICC, Union for International Cancer Control.

^a^: All of the patients were diagnosed with M1 due to positive lymph nodes other than the regional lymph nodes.

**SUPPLEMENTAL TABLE 3: Postoperative outcomes between the low and the high groups.**

|  | All  (n = 128) | Low (n = 24) | High  (n = 24) | P value |
| --- | --- | --- | --- | --- |
| Postoperative complications (Clavien-dindo classification grade ≥ 3), n | 24 (18.8) | 4 (16.7) | 6 (25.0) | 0.477 |
| Pancreatic fistula (ISGPF grade ≥ B), n | 23 (18.0) | 4 (16.7) | 4 (16.7) | > 0.999 |
| Postoperative hospital stay, day | 19 (6-73) | 18 (7-64) | 22 (6-73) | 0.307 |
| Adjuvant chemotherapy, n | 103 (83.1) | 21 (87.5) | 17 (73.9) | 0.286 |
| Recurrence within 6 months, n | 36 (28.1) | 3 (12.5) | 14 (58.3) | 0.001 |
| Recurrence, n | 98 (76.6) | 18 (75.0) | 22 (91.7) | 0.121 |
| Pattern of recurrence |  |  |  |  |
| Loco-regional recurrence, n | 54 (42.2) | 11 (45.8) | 9 (37.5) | 0.558 |
| Hepatic recurrence, n | 37 (28.9) | 4 (16.7) | 11(45.8) | 0.029 |
| Peritoneal recurrence, n | 19 (14.8) | 1 (4.2) | 5 (20.8) | 0.271 |
| Other distant recurrence, n | 21 (16.4) | 7 (29.2) | 3 (12.5) | 0.031 |

ISGPF, the International Study Group of Pancreatic Fistula; ISGPS, the International Study Group of Pancreatic Surgery.

**SUPPLEMENTAL TABLE 4: The top 30 pathways from a comparison between the non-tumor and the low group.**

| Rank | Canonical Pathways | p-value | -log(p-value) | #Molecules | Molecules |
| --- | --- | --- | --- | --- | --- |
| 1 | SPINK1 Pancreatic Cancer Pathway | 3.82E-08 | 7.42 | 12 | CELA2A, CELA3A, CLPS, CPA1, CPA2, CPB1, CPE, CTRB2, CTSB, KLK1, PRSS1, SPINK1 |
| 2 | BAG2 Signaling Pathway | 9.20E-08 | 7.04 | 13 | ANXA2, CTSB, HSP90AA1, HSPA1A/HSPA1B, HSPA8, HSPA9, PSMA5, PSMB5, PSMB7, PSMB8, PSMD2, PSME1, PSME2 |
| 3 | Aryl Hydrocarbon Receptor Signaling | 4.22E-07 | 6.37 | 16 | ALDH1A1, ALDH2, ALDH6A1, ALDH9A1, CTSD, GSTA2, GSTM3, GSTP1, HSP90AA1, HSP90AB1, HSP90B1, HSPB1, NFE2L2, PTGES3, TFF1, TGM2 |
| 4 | Clathrin-mediated Endocytosis Signaling | 7.81E-07 | 6.11 | 17 | ACTG2, ACTR3, ALB, APOE, ARF6, ARPC1B, CLTB, CLTC, CLU, DNM2, F2, HSPA8, ITGB1, LYZ, PDGFD, RAB11B, RPS27A |
| 5 | Protein Ubiquitination Pathway | 8.22E-07 | 6.09 | 20 | CRYAB, DNAJB11, HLA-B, HSP90AA1, HSP90AB1, HSP90B1, HSPA1A/HSPA1B, HSPA8, HSPA9, HSPB1, HSPE1, PSMA5, PSMB5, PSMB7, PSMB8, PSMD2, PSME1, PSME2, RPS27A, UBE2N |
| 6 | Sirtuin Signaling Pathway | 1.87E-06 | 5.73 | 20 | CPS1, GLUD1, H3-3A/H3-3B, IDH2, LDHA, LDHB, NDRG1, NDUFB4, NDUFS2, NDUFS5, NFE2L2, PGK1, PRKDC, SF3A1, SOD3, TSPO, TUBA4A, VDAC3, XRCC5, XRCC6 |
| 7 | Actin Cytoskeleton Signaling | 2.75E-06 | 5.56 | 18 | ACTG2, ACTN1, ACTN4, ACTR3, ARPC1B, F2, FLNA, FN1, IQGAP1, ITGB1, KNG1, MSN, MYL12A, MYL6, PDGFD, PFN1, RAC2, TLN1 |
| 8 | EIF2 Signaling | 3.40E-06 | 5.47 | 17 | ACTG2, EIF3F, PTBP1, RPL12, RPL23A, RPL24, RPL27, RPL4, RPL7, RPL7A, RPL9, RPLP0, RPLP2, RPS20, RPS25, RPS27A, RPS4X |
| 9 | Huntington's Disease Signaling | 3.40E-06 | 5.47 | 19 | CAPN1, CAPN2, CAPNS1, CLTB, CLTC, CTSD, DNM2, HSPA1A/HSPA1B, HSPA8, HSPA9, PSMA5, PSMB5, PSMB7, PSMB8, PSMD2, PSME1, PSME2, RPS27A, TGM2 |
| 10 | Xenobiotic Metabolism AHR Signaling Pathway | 3.50E-06 | 5.46 | 11 | ALDH1A1, ALDH2, ALDH6A1, ALDH9A1, GSTA2, GSTM3, GSTP1, HSP90AA1, HSP90AB1, HSP90B1, PTGES3 |
| 11 | Mitochondrial Dysfunction | 1.36E-05 | 4.87 | 14 | ACO1, ACO2, ATP5PO, COX4I1, COX5B, COX7A2, CYB5A, HSD17B10, NDUFB4, NDUFS2, NDUFS5, PRDX5, UQCRH, VDAC3 |
| 12 | Remodeling of Epithelial Adherens Junctions | 2.93E-05 | 4.53 | 9 | ACTG2, ACTN1, ACTN4, ACTR3, ARF6, ARPC1B, DNM2, IQGAP1, TUBA4A |
| 13 | Integrin Signaling | 3.30E-05 | 4.48 | 15 | ACTG2, ACTN1, ACTN4, ACTR3, ARF6, ARPC1B, CAPN1, CAPN2, CAPNS1, ITGB1, MYL12A, PFN1, RAC2, RHOC, TLN1 |
| 14 | Glycogen Degradation III | 3.90E-05 | 4.41 | 5 | GAA, PGM1, PGM2, PYGB, TYMP |
| 15 | Glutaryl-CoA Degradation | 1.08E-04 | 3.97 | 5 | ACAA2, ACAT1, CA1, HADHA, HSD17B10 |
| 16 | Unfolded protein response | 2.34E-04 | 3.63 | 9 | DNAJB11, HSP90B1, HSPA1A/HSPA1B, HSPA8, HSPA9, NFE2L2, P4HB, PDIA2, VCP |
| 17 | Caveolar-mediated Endocytosis Signaling | 4.25E-04 | 3.37 | 8 | ACTG2, ALB, COPG1, DNM2, FLNA, FLNB, HLA-B, ITGB1 |
| 18 | LXR/RXR Activation | 4.25E-04 | 3.37 | 10 | AHSG, ALB, AMBP, APOE, CLU, HPX, KNG1, LYZ, SERPINF1, TTR |
| 19 | FAT10 Signaling Pathway | 4.47E-04 | 3.35 | 7 | PSMA5, PSMB5, PSMB7, PSMB8, PSMD2, PSME1, PSME2 |
| 20 | Glycogen Degradation II | 4.25E-04 | 3.34 | 4 | PGM1, PGM2, PYGB, TYMP |
| 21 | TCA Cycle II (Eukaryotic) | 4.64E-04 | 3.33 | 5 | ACO1, ACO2, CS, DLST, MDH1 |
| 22 | Tryptophan Degradation III (Eukaryotic) | 4.64E-04 | 3.33 | 5 | ACAA2, ACAT1, CA1, HADHA, HSD17B10 |
| 23 | Virus Entry via Endocytic Pathways | 5.25E-04 | 3.28 | 9 | ACTG2, CLTB, CLTC, DNM2, FLNA, FLNB, HLA-B, ITGB1, RAC2 |
| 24 | Glycolysis I | 6.17E-04 | 3.21 | 5 | ALDOA, ENO2, GAPDH, PGK1, PKM |
| 25 | Gluconeogenesis I | 6.17E-04 | 3.21 | 5 | ALDOA, ENO2, GAPDH, MDH1, PGK1 |
| 26 | Polyamine Regulation in Colon Cancer | 6.41E-04 | 3.19 | 7 | PSMA5, PSMB5, PSMB7, PSMB8, PSMD2, PSME1, PSME2 |
| 27 | Oxidative Phosphorylation | 6.42E-04 | 3.19 | 9 | ATP5PO, COX4I1, COX5B, COX7A2, CYB5A, NDUFB4, NDUFS2, NDUFS5, UQCRH |
| 28 | Aldosterone Signaling in Epithelial Cells | 6.42E-04 | 3.19 | 11 | AHCY, CRYAB, DNAJB11, HSP90AA1, HSP90AB1, HSP90B1, HSPA1A/HSPA1B, HSPA8, HSPA9, HSPB1, HSPE1 |
| 29 | Regulation of Actin-based Motility by Rho | 9.83E-04 | 3.01 | 9 | ACTG2, ACTR3, ARPC1B, ITGB1, MYL12A, MYL6, PFN1, RAC2, RHOC |
| 30 | NRF2-mediated Oxidative Stress Response | 1.02E-03 | 2.99 | 13 | ACTG2, CBR1, DNAJB11, EPHX1, GSTA2, GSTM3, GSTP1, HSP90AA1, HSP90AB1, HSP90B1, NFE2L2, SOD3, VCP |

**SUPPLEMENTAL TABLE 5: The top 30 pathways from a comparison between the non-tumor and the high group.**

| Rank | Canonical Pathways | p-value | -log(p-value) | #Molecules | Molecules |
| --- | --- | --- | --- | --- | --- |
| 1 | SPINK1 Pancreatic Cancer Pathway | 6.41E-08 | 7.19 | 12 | CELA2A, CELA3A, CELA3B, CLPS, CPA1, CPA2, CPB1, CPE, CTSB, KLK1, PRSS1, SPINK1 |
| 2 | EIF2 Signaling | 1.05E-07 | 6.98 | 20 | ACTG2, EIF3F, PTBP1, RPL23A, RPL24, RPL27, RPL4, RPL7, RPL7A, RPL9, RPLP0, RPLP1, RPLP2, RPS10, RPS12, RPS20, RPS25, RPS27A, RPS4X, RPS6 |
| 3 | Aryl Hydrocarbon Receptor Signaling | 1.05E-07 | 6.98 | 17 | ALDH1A1, ALDH2, ALDH6A1, ALDH7A1, ALDH9A1, CTSD, GSTA2, GSTM3, GSTO1, GSTP1, HSP90AA1, HSP90B1, HSPB1, NFE2L2, PTGES3, TFF1, TGM2 |
| 4 | Clathrin-mediated Endocytosis Signaling | 2.30E-07 | 6.64 | 18 | ACTG2, ACTR3, ALB, APOE, ARF6, ARPC1B, ARPC4, CLTB, CLTC, CLU, DNM2, F2, HSPA8, ITGB1, LYZ, RAB5C, RPS27A, SERPINA1 |
| 5 | Actin Cytoskeleton Signaling | 2.44E-07 | 6.61 | 20 | ACTG2, ACTN1, ACTN4, ACTR3, ARPC1B, ARPC4, CRKL, F2, FLNA, FN1, IQGAP1, ITGB1, MSN, MYH11, MYH9, MYL12A, MYL6, PFN1, RAC2, TLN1 |
| 6 | BAG2 Signaling Pathway | 5.48E-07 | 6.26 | 12 | ANXA2, CTSB, HSP90AA1, HSPA1A/HSPA1B, HSPA8, PSMA2, PSMB5, PSMB7, PSMB8, PSMD2, PSME1, PSME2 |
| 7 | Remodeling of Epithelial Adherens Junctions | 5.48E-07 | 6.26 | 11 | ACTG2, ACTN1, ACTN4, ACTR3, ARF6, ARPC1B, ARPC4, DNM2, IQGAP1, RAB5C, TUBA4A |
| 8 | Integrin Signaling | 5.48E-07 | 6.26 | 18 | ACTG2, ACTN1, ACTN4, ACTR3, ARF1, ARF6, ARPC1B, ARPC4, CAPN2, CAPNS1, CRKL, ITGB1, MYL12A, PFN1, RAC2, RHOC, TLN1, VASP |
| 9 | Xenobiotic Metabolism AHR Signaling Pathway | 6.12E-07 | 6.21 | 12 | ALDH1A1, ALDH2, ALDH6A1, ALDH7A1, ALDH9A1, GSTA2, GSTM3, GSTO1, GSTP1, HSP90AA1, HSP90B1, PTGES3 |
| 10 | Glycolysis I | 4.89E-06 | 5.31 | 7 | ENO1, GAPDH, GPI, PGAM1, PGK1, PKM, TPI1 |
| 11 | Glycogen Degradation III | 6.24E-05 | 4.2 | 5 | GAA, PGM1, PGM2, PYGB, TYMP |
| 12 | RhoGDI Signaling | 7.00E-05 | 4.15 | 15 | ACTG2, ACTR3, ARHGDIA, ARHGDIB, ARPC1B, ARPC4, CD44, ITGB1, MSN, MYH11, MYH9, MYL12A, MYL6, RAC2, RHOC |
| 13 | Protein Ubiquitination Pathway | 7.25E-05 | 4.14 | 17 | CRYAB, HLA-B, HSP90AA1, HSP90B1, HSPA1A/HSPA1B, HSPA8, HSPB1, HSPE1, PSMA2, PSMB5, PSMB7, PSMB8, PSMD2, PSME1, PSME2, RPS27A, UBE2N |
| 14 | Regulation of Actin-based Motility by Rho | 7.32E-05 | 4.14 | 11 | ACTG2, ACTR3, ARHGDIA, ARPC1B, ARPC4, ITGB1, MYL12A, MYL6, PFN1, RAC2, RHOC |
| 15 | Caveolar-mediated Endocytosis Signaling | 7.88E-05 | 4.1 | 9 | ACTG2, ALB, COPG1, DNM2, FLNA, FLNB, HLA-B, ITGB1, RAB5C |
| 16 | Huntington's Disease Signaling | 7.88E-05 | 4.1 | 17 | CAPN2, CAPNS1, CLTB, CLTC, CTSD, DNM2, HSPA1A/HSPA1B, HSPA8, PSMA2, PSMB5, PSMB7, PSMB8, PSMD2, PSME1, PSME2, RPS27A, TGM2 |
| 17 | Acute Phase Response Signaling | 2.16E-04 | 3.67 | 13 | AHSG, ALB, AMBP, C3, CRABP2, F2, FN1, FTL, HNRNPK, ITIH2, RBP1, SERPINA1, SERPINA3 |
| 18 | Ethanol Degradation II | 2.17E-04 | 3.66 | 6 | ADH1B, ALDH1A1, ALDH2, ALDH7A1, ALDH9A1, DHRS4 |
| 19 | Noradrenaline and Adrenaline Degradation | 3.53E-04 | 3.45 | 6 | ADH1B, ALDH1A1, ALDH2, ALDH7A1, ALDH9A1, DHRS4 |
| 20 | Glycogen Degradation II | 5.18E-04 | 3.29 | 4 | PGM1, PGM2, PYGB, TYMP |
| 21 | LXR/RXR Activation | 5.18E-04 | 3.29 | 10 | A1BG, AHSG, ALB, AMBP, APOE, C3, CLU, LYZ, SERPINA1, VTN |
| 22 | FAT10 Signaling Pathway | 5.18E-04 | 3.29 | 7 | PSMA2, PSMB5, PSMB7, PSMB8, PSMD2, PSME1, PSME2 |
| 23 | Virus Entry via Endocytic Pathways | 7.48E-04 | 3.13 | 9 | ACTG2, CLTB, CLTC, DNM2, FLNA, FLNB, HLA-B, ITGB1, RAC2 |
| 24 | Epithelial Adherens Junction Signaling | 7.70E-04 | 3.11 | 11 | ACTG2, ACTN1, ACTN4, ACTR3, ARPC1B, ARPC4, IQGAP1, MYH11, MYH9, MYL6, TUBA4A |
| 25 | Gluconeogenesis I | 7.70E-04 | 3.11 | 5 | ENO1, GAPDH, GPI, PGAM1, PGK1 |
| 26 | Phagosome Maturation | 7.70E-04 | 3.11 | 11 | CTSB, CTSD, CTSE, CTSG, HLA-B, HLA-DRB1, LAMP1, MPO, RAB5C, RAC2, TUBA4A |
| 27 | Xenobiotic Metabolism CAR Signaling Pathway | 7.95E-04 | 3.1 | 15 | ALDH1A1, ALDH2, ALDH6A1, ALDH7A1, ALDH9A1, GSTA2, GSTM3, GSTO1, GSTP1, HSP90AA1, HSP90B1, SOD3 |
| 28 | Polyamine Regulation in Colon Cancer | 7.95E-04 | 3.1 | 7 | PSMA2, PSMB5, PSMB7, PSMB8, PSMD2, PSME1, PSME2 |
| 29 | Xenobiotic Metabolism Signaling | 9.57E-04 | 3.02 | 15 | ALDH1A1, ALDH2, ALDH6A1, ALDH7A1, ALDH9A1, FTL, GSTA2, GSTM3, GSTO1, GSTP1, HSP90AA1, HSP90B1, NFE2L2, PTGES3, SOD3 |
| 30 | Sirtuin Signaling Pathway | 1.04E-03 | 2.98 | 15 | H3-3A/H3-3B, IDH2, LDHA, LDHB, NDRG1, NDUFB4, NFE2L2, PGAM1, PGK1, SF3A1, SLC25A5, SOD3, TUBA4A, VDAC3, XRCC6 |
